# To Study the Correlation of Maternal Serum Vitamin D Levels and Infant Serum Vitamin D Levels With Infant Birth Weight: A Single-Centre Experience From the Bundelkhand Region, India

**DOI:** 10.7759/cureus.68696

**Published:** 2024-09-05

**Authors:** Mayank Singh, Hema Shobhane, Kapil Tiwari, Shristy Agarwal

**Affiliations:** 1 Pathology, Maharani Laxmi Bai Medical College, Jhansi, IND; 2 Obstetrics and Gynecology, Maharani Laxmi Bai Medical College, Jhansi, IND

**Keywords:** birth weight, low birth weight, maternal nutrition, newborn, vitamin d

## Abstract

Background: Vitamin D deficiency during pregnancy can have severe effects on both the mother and the newborn child. The main aim of this study was to assess the impact of maternal vitamin D levels on the birth weight of the newborn by analysing the vitamin D levels in pregnant women at full term and their newborn.

Material and methods: The cross-sectional, hospital-based study was conducted with 150 consecutive women in labour presenting with a singleton term pregnancy at a large tertiary centre in the Bundelkhand region, India. Maternal and infant blood samples were obtained at the time of delivery. Umbilical cord blood was collected from infants, while maternal venous blood was drawn simultaneously. All relevant data were gathered, including the assessment of 25-hydroxy vitamin D3 levels in both mother and infant. The birth weight of the infant was measured, and statistical analysis was performed to find an association between maternal vitamin D level to birth weight and vitamin D level of the infant.

Result: Most pregnant women had low vitamin D levels in this study. The results revealed a significant positive correlation between maternal serum vitamin D levels and infant birth weight (p < 0.001), suggesting that lower maternal vitamin D levels were associated with low birth weight in infants. Additionally, infant serum vitamin D levels showed a positive correlation with maternal vitamin D levels (p < 0.001), indicating that higher maternal vitamin D levels tend to have infants with higher vitamin D levels at birth.

Conclusion: These findings suggest a potential correlation of maternal vitamin D status to birth weight and vitamin D level of newborns, and further research is needed to confirm and better understand this relationship. Additionally, other factors such as maternal nutrition, genetics, lifestyle factors, and environmental influences may contribute to birth weight outcomes.

## Introduction

A woman during her pregnancy is required to consume a sufficient amount of vitamin D for her optimal health and for adequate growth of the developing fetus [1]. The increased vitamin D requirements during pregnancy can be met through increasing dietary intake, supplements, and sufficient sun exposure [2]. In a tropical country such as India, vitamin D deficiency is unexpected as there is abundant sunshine around the year. Vitamin D levels during pregnancy play a pivotal role in maternal health and fetal development, along with optimal neonatal outcomes, as well as the long-term health of the child [3-6].

Serum vitamin D levels doubled from pre-pregnant levels at around 10-12 weeks of pregnancy, continue to rise, and reach a peak level during the third trimester [7]. The resulting increase in the active form of vitamin D points to a potential role for vitamin D in supporting maternal health [8].

Studies have shown a link between inadequate maternal vitamin D levels during pregnancy to a range of potential complications, encompassing preeclampsia, gestational diabetes, cesarean section, infections, low birth weight, and preterm delivery [9,10].

Preterm birth is associated with an increased risk of infant mortality, as well as long-term neurodevelopmental disabilities, including cerebral palsy, developmental delays, learning disabilities, and behavioral disorders [11,12]. The vitamin D status of the fetus and newborn is primarily influenced by the mother's vitamin D status [13]. To minimize the potential risks of vitamin D deficiency and its associated complications, such as low birth weight, pregnant women are typically advised to ensure adequate vitamin D intake through dietary sources, supplementation, and safe sun exposure. However, in India, screening for vitamin D deficiency is not included in standard antenatal care. Therefore, further research is needed to gather data from diverse regions within the country to comprehensively assess the prevalence of this issue and develop effective preventive measures.

This study aimed to assess the association between maternal vitamin D levels on neonatal serum vitamin D levels and birth weight of full-term infants at a tertiary care centre in the Bundelkhand region of India.

## Materials and methods

An analytical cross-sectional study was conducted in the Department of Pathology, MLB Medical College, Jhansi, in association with the Department of Obstetrics and Gynecology, MLB Medical College, Jhansi, from Nov 2023 to July 2024. The study was conducted on 150 mothers and their newborns. Approval was obtained from the Institute Ethics Committee (Human Studies) of MLB Medical College, Jhansi, and all participants gave informed consent before participating in the study. There was no additional cost to our participants.

Inclusion criteria: The target population included all otherwise normal pregnant women presenting at full term (≥ 37 weeks of gestation) providing consent for inclusion in the study and their infants, regardless of route of delivery (normal vaginal delivery/lower segment cesarean section).

Exclusion criteria: Mothers with conditions that are known to affect infant birth weight, such as pre-eclampsia, eclampsia, insulin-dependent diabetes mellitus, systemic or chronic diseases, hematologic disorders, medication use, twin pregnancies, or substance abuse, were not included in the study. In addition, newborns with congenital malformations or TORCH (toxoplasmosis, rubella cytomegalovirus, herpes simplex, and HIV) infections were excluded.

For the selected participants, the sex of the newborn, birth weight and height, gestational age, mode of delivery, and the age, parity, and educational level of the mother were documented. All newborns were physically examined and anthropometrically measured, and the findings were recorded according to the established protocol.

Data collection methods: 5 mL of maternal blood was collected in a plain vial under sterile conditions to assess vitamin D levels. Following delivery and cord clamping, an additional 3 mL blood sample was obtained from the umbilical cord in a separate plain vial to determine the newborn's vitamin D status. Both samples were then analyzed using the Abbott Architect i1000 SR (Abbott Core Laboratory, Green Oaks, IL). This study defined vitamin D deficiency as serum 25(OH)D levels below 20 ng/dL (50 nmol/L), following criteria established by the US Endocrine Society.

Birth weight categorization followed the World Health Organization (WHO) definitions, with low birth weight (LBW) defined as less than 2,500 grams (including 2,499 grams) at term birth. In this study, maternal serum 25(OH)D levels were categorized into three groups based on established criteria [14]: vitamin D deficiency: levels below 20 ng/mL (or 50 nmol/L); vitamin D insufficiency: levels between 20 and 29.9 ng/mL (or 50 and 75 nmol/L); and vitamin D sufficiency: levels at or above 30 ng/mL (or 75 nmol/L).

Statistical analysis

The Statistical Product and Service Solutions (SPSS, version 27.0; IBM SPSS Statistics for Windows, Armonk, NY) software package was used for statistical analysis. Data are presented as mean ± standard deviation for continuous variables and n% for categorical variables. The chi-square test was used for the analysis of categorical variables. One-way ANOVA and Pearson's correlation coefficient were used where appropriate to examine whether maternal serum 25(OH)D levels were correlated with neonatal birth weight. The level of significance was considered to be p < 0.05.

## Results

Table 1 shows the general characteristics of maternal parameters. Most of the cases (56.67%) were below 25 years of age and the mean age of mothers was 23.93 ± 3.198 years. Of the 150 cases, 84 (56.0%) were primipara. The mean weight of the mothers was 55.07 ± 7.338 kg, and the mean Hb level was 10.05 ± 1.627 gm/dL. Vitamin D level was deficient (<20 ng/mL) in 83 mothers, followed by 39 cases with inadequate vitamin D level (20-29.9 ng/mL), and only 28 (18.67%) had adequate vitamin D level (i.e., > 30 ng/mL). Only five (3.33%) individuals were receiving vitamin D supplementation. Most of the cases (61.33%) were delivered by lower cesarean section compared to normal delivery (38.67%).

**Table 1:** General characteristics of maternal parameters

Parameters	Value	No.	%
Age (in years)	<25	85	56.67
	25-30	60	40.00
	>30	5	3.33
	Mean ± SD	23.93 ± 3.198	
Parity	Primigravidae	84	56.0
	Multigravida	66	44.0
Weight (kg)	Mean ± SD	55.07±7.338	
Gestational age (weeks)	Mean ± SD	38.11±1.606	
Hb (gm/dL)	Mean ± SD	10.05 ± 1.627	
Vitamin D level	Deficiency (<20 ng/mL)	83	55.33
	Insufficiency (20-29.9 ng/mL)	39	26.00
	Sufficiency (>30 ng/mL)	28	18.67
	Mean ± SD	19.38 ± 10.216	
Vitamin D supplementation	Yes	5	3.33
	No	145	96.67
Route of delivery	Normal Vaginal	58	38.67
	LSCS	92	61.33

The mean birth weight of newborns whose mothers had vitamin D deficiency, vitamin D insufficiency, and vitamin D sufficiency was 2,162.65 ± 296.714 g, 2,798.718 ± 329.770 g, and 3,357.143 ± 358.421 g, respectively. The difference was highly statistically significant with ANOVA (p < 0.001). The mean serum vitamin D levels of the newborns were 8.19 ± 4.37 ng/mL, 18.05 ± 3.73 ng/mL, and 23.93 ± 6.50 ng/mL for mothers whose vitamin D levels were deficient (<20 ng/mL), insufficient (20-29.9 ng/mL), and sufficient (>30 ng/mL), respectively. Using the ANOVA test, the difference was statistically highly significant with p < 0.001 (Table 2).

**Table 2:** Correlation of maternal vitamin D levels to newborn birth weight and their vitamin D level ^1^=ANOVA, *=Significant (p<0.05)

Newborn parameters	Maternal Vitamin D Level (ng/mL)			^1^p-Value
	Deficiency (<20 ng/mL) (n=83)	Insufficiency (20-29.9 ng/mL) (n=39)	Sufficiency (>30 ng/mL) (n=28)	
	Mean ± SD	Mean ± SD	Mean ± SD	
Newborn Birth weight (gm)	2162.65 ± 296.714	2798.718 ± 329.770	3357.143 ± 358.421	<0.001^*^
Newborn Vitamin D level (ng/dL)	8.19 ± 4.37	18.05 ± 3.73	23.93 ± 6.50	<0.001^*^

A total of 80 (96.39%) newborns were born to low birth weight (i.e., <2,500 gm birth weight) from 83 women whose maternal vitamin D level was deficient (<20 ng/mL), while 35 (97.44%) newborns born to be normal birth weight (i.e., >2,500 gm) from mothers whose maternal vitamin D level was insufficient (20-29.9 ng/mL). All mothers with sufficient maternal vitamin D levels (>30 ng/mL) (n=28) gave birth to newborns of 100% normal birth weight. On statistical analysis, p is <0.001, which is highly significant. From the above data, we can find a significant association between maternal vitamin D deficiency and an increased risk of low birth weight (Table 3).

**Table 3:** Association of maternal vitamin D levels by newborn birth weight ^1^=Chi-square test, *=Significant (p<0.05)

Maternal Vitamin D Level (ng/mL)	No. of neonates born				^1^p-value
	Low birth weight (<2500 gm)		Normal birth weight (>2500 gm)		
	No.	%	No.	%	
Deficiency (<20 ng/mL) (n=83)	80	96.39	3	3.61	< 0.001^*^
Insufficiency (20-29.9 ng/mL) (n=39)	4	2.56	35	97.44	
Sufficiency (>30 ng/mL) (n=28)	0	0.00	28	100	
Total	84	56.0	66	44.0	

**Figure 1:**
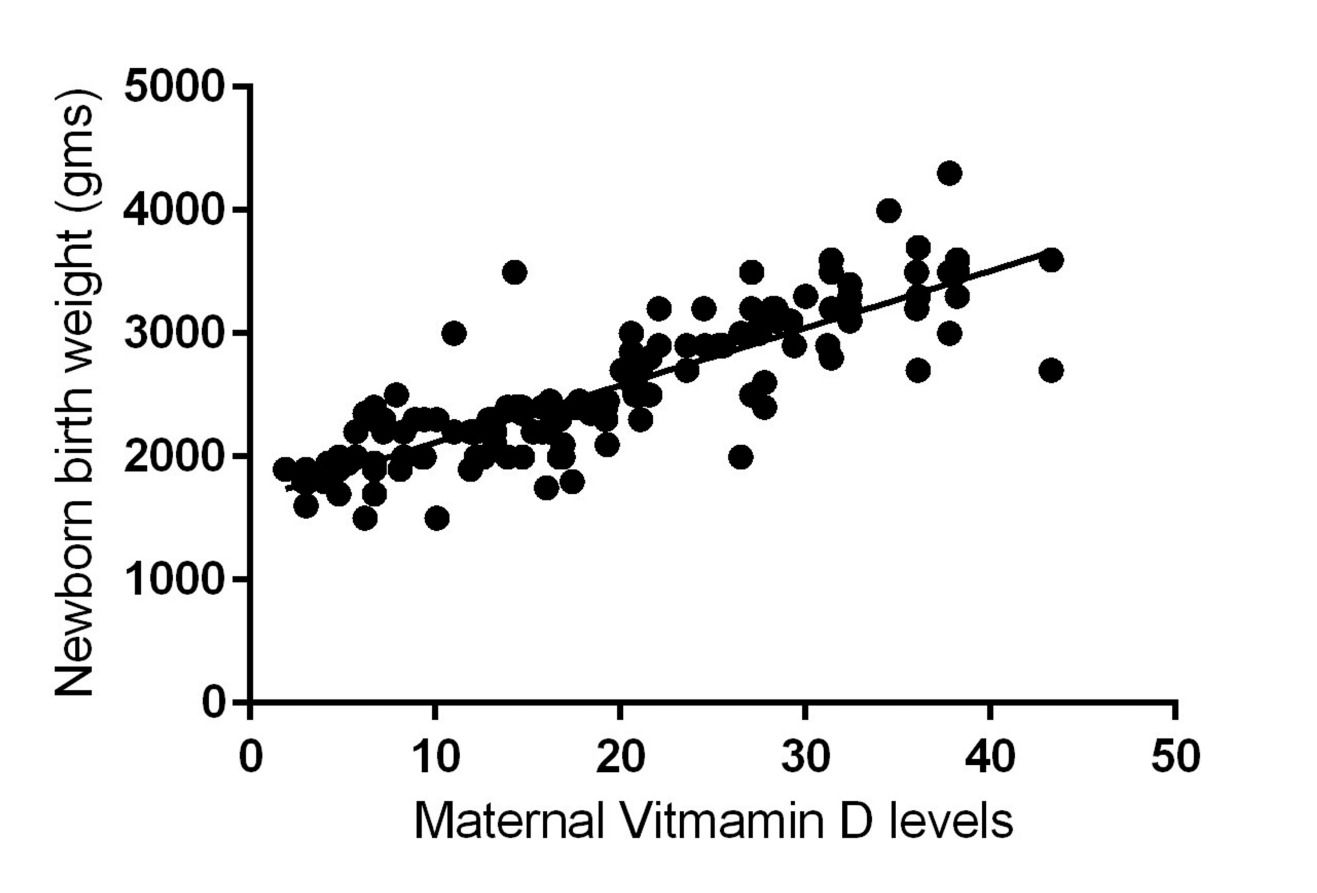
Graph illustrating the relationship between maternal serum 25(OH)D levels during pregnancy and the birth weight of their newborns was derived from data collected from 150 pregnant women The figure demonstrates a strong positive correlation between these two factors. The statistical analysis confirms this association, with a correlation coefficient (r) of 0.8371 and a p-value <0.001, indicating a highly significant relationship.

Analysis of the relationship between maternal serum 25(OH)D levels during pregnancy and newborn birth weight, as depicted in Figure 1, revealed a positive correlation. This correlation was statistically significant, with a correlation coefficient (r) of 0.8371 and a p-value <0.001. Furthermore, the analysis identified a threshold of approximately 18 ng/mL for maternal serum 25(OH)D levels. Below this threshold, maternal 25(OH)D levels emerged as a significant predictor of newborn birth weight. Linear regression analysis was employed to further investigate the relationship between these two variables.

## Discussion

Vitamin D, a fat-soluble secosteroid, is synthesized endogenously when ultraviolet B radiation from sunlight catalyzes the conversion of 7-dehydrocholesterol in the skin to previtamin D3, which subsequently undergoes thermal isomerization to form vitamin D3 (cholecalciferol) [15]. Alternatively, it can be obtained exogenously from dietary sources such as fatty fish, fortified foods, or supplements, in the form of either vitamin D2 (ergocalciferol) from plants or vitamin D3 from animal-derived sources [16]. Its primary physiological function revolves around the regulation of calcium and phosphate homeostasis, crucial for bone mineralization, through its interaction with the vitamin D receptor in various target tissues [17]. Additionally, vitamin D plays a role in modulating immune function, cellular growth, and neuromuscular function. However, its levels are subject to variation influenced by factors including latitude, seasonal changes, skin pigmentation, age, dietary intake, and individual differences in the capacity for endogenous synthesis. Understanding these factors is essential for optimizing health outcomes and preventing deficiencies or excesses of this vital nutrient [18].

Pregnant women, especially those living in regions with cold winters, are particularly vulnerable to vitamin D deficiency [19]. Approximately 50% of pregnant African American women in the United States have vitamin D insufficiency, defined as levels below 37.5 nmol/L [20], compared to 30% of pregnant Caucasian women. There is a positive correlation between maternal 25(OH)D levels and levels in placental veins, suggesting that calcidiol readily crosses the placental barrier [21]. Therefore, fetal vitamin D levels are directly dependent on maternal levels [22]. Studies comparing infants in countries that supplement milk products with vitamin D to those in countries that do not show lower mineral levels in the latter group, as assessed by dual-energy x-ray absorptiometry scans [23].

The current study examined the relationship between maternal and newborn vitamin D concentrations and infant birth weight at a research center in the Bundelkhand region, focusing on environmental, nutritional, and genetic variables affecting vitamin D levels. The findings revealed a robust positive correlation between maternal blood 25(OH)D levels and infants' birth weights. Specifically, low maternal vitamin D levels (<20 ng/mL) were significantly associated with low birth weights, while sufficient levels (>30 ng/mL) correlated with normal birth weights, underscoring the importance of adequate vitamin D throughout pregnancy for infant health.

Similarly, Motlagh et al. found that mothers had an average weight of 55.07 ± 7.34 kg and an average hemoglobin level of 10.05 ± 1.63 g/dL, suggesting these characteristics reflect broader maternal health trends across various groups [24]. In contrast, Sathish et al. reported a lower mean maternal age compared to our study, with a significant portion of mothers having vitamin D levels below recommended thresholds [24].

Studies by Sangle et al. [25] and Bassir et al. [26] revealed a prevalent occurrence of hypovitaminosis D among pregnant women, leading to decreased levels in newborns. These studies highlight concerning trends in maternal health, particularly in regions such as India where inadequate consumption of vitamin D-rich foods exacerbates the issue, compounded by the limited availability of fortified foods. Further research by Sangle et al. [25] and Kaludjerovic et al. revealed that hypovitaminosis D in pregnant women increases the risk of negative outcomes, such as preeclampsia, gestational diabetes, bacterial vaginosis, and low birth weight infants. These findings emphasize the critical importance of ensuring pregnant women receive adequate vitamin D to mitigate these risks and promote optimal health for both mother and fetus [26,27].

Additionally, the current findings revealed a significant correlation between maternal vitamin D levels and neonatal birth weight. Among mothers with vitamin D deficiency (<20 ng/mL), 96.39% of neonates had low birth weight, indicating a significant correlation (p < 0.00001). In the insufficiency group (20-29.9 ng/mL), the difference was not significant, with 2.56% of neonates having low birth weight. Among those with sufficient vitamin D levels (>30 ng/mL), none had low birth weight, suggesting a non-significant relationship.

Similarly, Khalessi et al. [28] found significant links between maternal vitamin D deficiency and neonatal health. Neonates with smaller head circumferences were consistently born to vitamin D-deficient mothers (p = 0.007), while neonates born via normal vaginal delivery had lower rates of maternal vitamin D deficiency compared to cesarean section deliveries (p = 0.027). Although no significant differences were noted in maternal vitamin D levels concerning neonatal height (p = 0.054), vitamin D-deficient mothers tended to be older (p = 0.031) [28,29].

This work greatly enhances its understanding of the correlation between maternal vitamin D levels and the weight of newborns, providing useful insights to the current body of research. The study establishes a strong connection between the vitamin D levels of mothers and the weight of their infants, using rigorous methods and thorough data analysis. The results highlight the possible negative effects on health caused by insufficient levels of vitamin D during pregnancy, underlining the crucial importance of mother nutrition in the development of the fetus.

These findings are now of even greater relevance as they emphasize how maintaining adequate levels of vitamin D in pregnant mothers could improve health outcomes both for the mother and the baby. Vitamin D is essential for many physiological processes, such as maintaining calcium balance and promoting skeletal development. These processes are especially important during pregnancy when there is rapid growth and development of the fetus. The research findings indicate that insufficient amounts of vitamin D in pregnant women might result in below-average fetal development, which could raise the possibility of unfavorable delivery outcomes.

Moreover, the study's insistence on additional longitudinal research is crucial for several reasons. Longitudinal studies may provide valuable information on the cause-and-effect connections between a mother's vitamin D levels and the weight of her baby at delivery. This allows researchers to better understand the underlying processes and pinpoint possible opportunities for intervention. Comprehending these pathways is crucial for devising precise solutions for effectively addressing vitamin D inadequacy in pregnant populations.

Furthermore, longitudinal research could assist in the identification of possible confounding factors and sources of bias, hence enhancing the validity and reliability of the results. Through the longitudinal monitoring of maternal vitamin D levels and birth outcomes, researchers may more accurately consider variables such as seasonal fluctuations, food consumption, and lifestyle factors that can impact the association between vitamin D status and birth weight.

Furthermore, longitudinal studies enable the analysis of extended health consequences that go beyond birth weight, such as childhood growth and development, immunological function, and vulnerability to chronic illnesses. A comprehensive approach is necessary for understanding completely the range of health consequences linked to insufficient levels of vitamin D in expectant mothers throughout pregnancy.

In general, this research highlights the significance of maternal nutrition, particularly sufficient consumption of vitamin D, in enhancing positive outcomes throughout pregnancy. The study emphasizes the need for further research and intervention techniques, offering useful insights that could possibly guide the development of public health policies and clinical practices to enhance the well-being of mothers and children.

## Conclusions

The present study has shown a positive correlation between the health status of women and newborns with reference to vitamin D levels in both mothers and newborns in the central part of India. Accordingly, it was found that a relatively higher proportion of vitamin D deficiency is seen among women, an important nutrient involving the health of both mother and baby. Another critical part of the research is that the finding reflects a significant correlation between the vitamin D levels in a mother's blood during pregnancy and the vitamin D status reflected in the baby's blood at birth. In many ways, therefore, the vitamin D status of the mother significantly affects her child's vitamin D status. The study also showed some potential signs of an association between maternal and infant vitamin D levels with birth weight. This can mean that a mother's vitamin D levels during pregnancy affect the child's birth weight, probably through mechanisms that affect intrauterine development or growth.

Although these results are very encouraging, it would be prudent to consider that several other factors could also affect birth weight outcomes. It might involve everything from general nutritional status regarding heredity on the mother's part down to individual choices made in lifestyle through to environmental factors. Further investigations are thus called for to clarify this association and enrich the scientific knowledge about maternal vitamin D concentration concerning birth weight in infants. In contrast, systematic screening of pregnant women for vitamin D deficiency, coupled with the appropriate intervention programs, might contribute much towards health outcomes for mothers and neonates, including outcomes associated with birth weight. Such a proactive approach may be relevant in promoting healthier pregnancies and better outcomes for infant health.
